# A qualitative study of the determinants of dieting and non-dieting approaches in overweight/obese Australian adults

**DOI:** 10.1186/1471-2458-12-1086

**Published:** 2012-12-18

**Authors:** Stuart Leske, Esben Strodl, Xiang-Yu Hou

**Affiliations:** 1 School of Psychology and Counselling, Queensland University of Technology, Victoria Park Road, Kelvin Grove, QLD, 4059, Australia; 2 School of Public Health and Social Work, Queensland University of Technology, Victoria Park Road, Kelvin Grove, QLD, 4059, Australia

**Keywords:** Diet, Dieting, Non-dieting, Qualitative, Grounded theory, Overweight, Obesity, Adults, Weight

## Abstract

**Background:**

Dieting has historically been the main behavioural treatment paradigm for overweight/obesity, although a non-dieting paradigm has more recently emerged based on the criticisms of the original dieting approach. There is a dearth of research contrasting why these approaches are adopted. To address this, we conducted a qualitative investigation into the determinants of dieting and non-dieting approaches based on the perspectives and experiences of overweight/obese Australian adults.

**Methods:**

Grounded theory was used inductively to generate a model of themes contrasting the determinants of dieting and non-dieting approaches based on the perspectives of 21 overweight/obese adults. Data was collected using semi-structured interviews to elicit in-depth individual experiences and perspectives.

**Results:**

Several categories emerged which distinguished between the adoption of a dieting or non-dieting approach. These categories included the focus of each approach (weight/image or lifestyle/health behaviours); internal or external attributions about dieting failure; attitudes towards established diets, and personal autonomy. Personal autonomy was also influenced by another category; the perceived knowledge and self-efficacy about each approach, with adults more likely to choose an approach they knew more about and were confident in implementing. The time perspective of change (short or long-term) and the perceived identity of the person (fat/dieter or healthy person) also emerged as determinants of dieting or non-dieting approaches respectively.

**Conclusions:**

The model of determinants elicited from this study assists in understanding why dieting and non-dieting approaches are adopted, from the perspectives and experiences of overweight/obese adults. Understanding this decision-making process can assist clinicians and public health researchers to design and tailor dieting and non-dieting interventions to population subgroups that have preferences and characteristics suitable for each approach.

## Background

The World Health Organisation has declared obesity as a global epidemic, predicting that there will be 2.3 billion overweight and over 700 million obese adults by 2015 [1]. Australia’s obesity rates are among the highest in the OECD and have steadily risen during the past 30 years [2], with 28.3% of adults obese and 35% overweight in 2011–12 [3]. Although excess weight increases the risk of type II diabetes, some cancers, cardiovascular diseases, asthma, gallbladder disease, osteoarthritis, and chronic back pain [4], these health risks can be reduced through modest weight loss and prevention of weight regain in overweight and obese adults [5].

These well documented physical health consequences and the associated benefits of weight loss have provided impetus to public health approaches (at the population level) and clinical guidelines (at an individual level) which warn the general public of the health risks of overweight/obesity and promote healthy eating and physical activity as preventative strategies to lose weight [6,7]. Perhaps as a result, weight control efforts appear to be commonplace, with reports that a third [8,9] to two-thirds [10] of adults in Western populations are trying to lose weight or avoid weight gain. The literature described below reveals a tension in these approaches between the promotion of weight loss or the promotion of increased healthy behaviour change as an end in itself to manage overweight and obesity successfully and sustainably.

### Responses to public health approaches for overweight and obesity

Despite the popularity of risk-based campaigns, knowledge of the health risks of excess weight does not appear to translate successfully to increased intentions, efforts, or success in losing weight [11,12]. Obese individuals also perceive that these public health messages a) overemphasise the physical health risks associated with obesity while underemphasising the social (e.g., stigma) and psychological (e.g., distress) dimensions, b) focus too much on weight measurement and weight loss, c) neglect discussion of the day-to-day management of obesity-related comorbidities, d) avoid discussion of the risk behaviours which lead to obesity such as unhealthy eating and sedentary lifestyles, and e) do not convey information about the complex aetiology of obesity and thus the need for comprehensive solutions [6]. This absence of both aetiological information and the promotion of complex, evidence-based solutions was perceived by these participants to push obese individuals towards extreme, unproven, expensive, short-lived solutions which were ultimately perceived to be unable to improve health and wellbeing. The emphasis on personal responsibility encapsulated in these public health messages also appeared to produce guilt, blame, shame, and a feeling of failure in those who were obese [6].

In qualitative studies, obese individuals have suggested that the focus of public health messages should shift from body mass index and weight to a broader focus on health and promotion of the benefits of a healthy lifestyle [6,13]. Large-scale quantitative data complements this; a nationally representative sample of American adults recently indicated that obesity-related public health messages focused on healthy eating and multiple health behaviours were perceived most favourably [14]. Notably, messages were also perceived most positively when they did not mention the word ‘obesity’ at all and focused on making healthy behaviour change without mentioning weight loss.

### Clinical guidelines for treatment of overweight and obesity

Public health approaches are complemented by evidence-based clinical guidelines for the management of adult obesity. Several of these guidelines [7,15-18] recommend multicomponent programs focusing on physical activity, dietary change, and behavioural components to lose weight or prevent weight gain/regain. In some guidelines [7], focusing on behaviour change and improved health appear to be framed as a means to an end (weight loss) rather than an end in itself.

### Treatment paradigms for overweight and obesity

These tensions have ultimately resulted in the emergence of a new non-dieting paradigm in the treatment of overweight/obesity, typically used by those in the eating disorders field [19]. Historically, the main behavioural treatment paradigm for weight loss used by professionals in the obesity field has been dieting [19], which can be defined as: “the intentional and sustained restriction of caloric intake for the purpose of reducing body weight or changing body shape” [20]. In hospital- and university-based settings, this paradigm takes the form of low- or very-low-calorie diets [21] and behavioural or cognitive-behavioural weight reduction strategies [22]. In the wider community, commercial weight loss programs and do-it-yourself diets are the dominant forms of this paradigm [23]. Unfortunately, there appears to be little support for the notion that dieting produces lasting weight loss or health benefits when attempted in isolation [24]. While moderate weight loss can be achieved in multicomponent programs [25], it is often regained over time, and some trials suffer from the lack of long-term follow-up required to document this weight regain [22]. Despite the equivocal support for this paradigm, diets appear to be popular with the general public, as recent data [10,26] suggests that approximately a third of adults may adopt what might be construed as a diet (liquid diet supplements, commercial weight loss programs, or special diets).

During the 1990s, the dieting paradigm was criticised for its inability to achieve long-term weight loss [27,28]. Critics argued that the weight-centred paradigm also contributed to food and body preoccupation, weight cycling, lower self-esteem, eating disorders, and weight stigmatisation [29-31]. An alternative non-dieting paradigm emerged, focusing instead on body acceptance, health behaviours, and health outcomes, without a focus on weight loss [32]. Other tenets of this approach included eating intuitively (i.e., relying on internal cues indicating hunger and satiation), rather than dietary restriction [33], and enjoying physical activity in contrast to participating in structured exercise [32]. A 2011 review [32] of the six existing randomised controlled trials testing non-dieting approaches observed clinically and statistically significant improvements in blood pressure, blood lipids, physical activity, eating disorder pathology, mood, self-esteem, and body image. The authors also responded to concerns that the exclusion of weight control messages would lead to weight gain by noting that no weight gain resulted from any of the ten published non-dieting trials.

Given that approximately 30-40% of weight-concerned adults [10,26] report trying to manage weight using what may be construed as a diet (liquid diet supplements, commercial weight loss programs, or a special diet), non-dieting is potentially a prevalent alternative approach to weight management. There appear to be unresolved issues with the adoption of this paradigm though, including the lack of empirical evaluation for non-dieting approaches; misleading conclusions based on inadequate evidence about the harmful effects of dieting, weight cycling, and excess weight; the underemphasis on reducing medical risk, and the minimisation of the environmental aspects of weight regulation [19]. In addition, non-dieting studies have been conducted with mostly white, female, overweight and obese subjects with a history of chronic dieting or binge eating [34], limiting the generalisability of the results.

There has been limited research contrasting why dieting and non-dieting approaches are adopted from the perspective of overweight/obese adults. Greater attention needs to be dedicated to non-dieting approaches [13], given that obese individuals prefer positive gain-framed health messages about lifestyle change rather than a focus on weight loss [6,13,35,36]. It is important to study these determinants not only to inform clinical practitioners and public health researchers about why each approach is taken, but to establish who may be best suited to different approaches [19]. In light of this, our research question was: What are the determinants of dieting and non-dieting approaches as described by overweight/obese adults? To ensure our model of these determinants was based on the experiences and perspectives of overweight/obese adults, we used grounded theory inductively to analyse data and generate the model. Our article therefore reports on this model of determinants.

## Methods

### Data collection

Data collection was approved by QUT's University Human Research Ethics Committee, and overweight/obese adults were recruited through recruitment flyers at the university’s health clinics, snowball sampling, and a radio broadcast. Purposive sampling of overweight/obese adults was used as a recruitment strategy to provide rich and varied data relevant to the research question [37,38]. Before participating, respondents read a detailed participant information sheet and provided informed consent. Interviews were chosen to collect data because the topic was considered sensitive and interviews seemed to be a better format to elicit in-depth individual experiences and perceptions.

Grounded theory [39] was used inductively to collect and analyse data. Emerging concepts were grounded in the data and the constant comparative method was used to systematically look for similarities and differences between participants’ experiences. Data collection and analysis occurred simultaneously to allow us to sample theoretically based on incoming data. This incoming data indicated that strong negative attitudes towards dieting might encourage non-dieting approaches, so we interviewed one formerly overweight adult who explicitly rejected dieting approaches to better understand these determinants. We sampled theoretically from a local lifestyle change clinic towards the end of data collection when it became apparent that ‘lifestyle change’ was the term being used to denote non-dieting approaches. Two adults from the clinic who explicitly rejected dieting approaches were interviewed.

The first author, a male PhD student, conducted all interviews between July 2010 and January 2011 using a guided discussion format recommended by grounded theorists [40]. Interviews were conducted either in face-to-face format at the Brisbane-based university or by phone for adults who had heard the radio broadcast and wanted to participate but lived remotely. Interviews began by asking participants about: a) their experiences with being overweight and losing weight; b) the meaning of weight; c) the meaning of food and exercise to them; and d) the impact (if any) their weight had on their self-identity and well-being. These domains were used as a “start list” [41] to segment the data into rationally derived domains, which were amended to more adequately reflect emerging concepts as data collection continued. In total, 21 interviews were conducted, ranging in duration from 40 minutes to 2 hours and 30 minutes. Interviews were transcribed verbatim by the first author, and transcripts were not linked with participant consent forms to ensure anonymity. Data collection continued until no new relevant themes appeared to be emerging.

### Participants

Inclusion criteria included a body mass index (BMI) above 25, and aged between 20 and 70. Exclusion criteria included a current pregnancy and any self-reported severe mental health problems (e.g., schizophrenia, severe depression, or diagnosed personality disorders). Six males and 15 females participated, and ages ranged from 24–69 years with a mean of 46.3 (*SD* = 12.87). Body mass index ranged from 24–50, with one adult previously overweight (BMI 24); five currently overweight (BMI 25–29.9); five class I obese (BMI 30–34.9); five class II obese (BMI 35–39.9), and five class III obese (BMI ≥40). Eight participants had completed postgraduate education; nine had completed undergraduate studies; three had completed technical and further education (TAFE), and one had completed high school. Ten adults dropped out of the study prior to being interviewed: several were unresponsive to emails, one thought help was being offered, one was instructed to call by another person and did not return the consent form, and one decided that they felt uncomfortable talking about the topic after initially indicating interest in participating.

### Data analysis

Consistent with recommendations [40], data analysis and the writing of memos began after the first interview. During the interview phase, the first author began to open code the data into higher- and lower-order concepts, delineating the properties and dimensions of each concept. NVivo 8.0 Qualitative Data Analysis [42] was used to organise themes. Axial coding related concepts to each other and diagrams were drawn to indicate the influences between each of the concepts and visualise the initial model. Selective coding was used to integrate the developed categories and identify the core variable. Codes and themes were triangulated between the first, second, and third authors to enhance the validity of open, axial, and selective coding phases.

## Results

**Figure 1:**
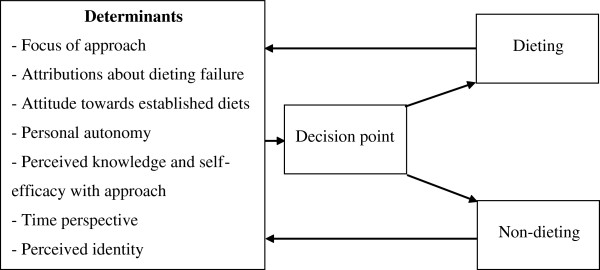
**Determinants of decision to adopt dieting or non**-**dieting approaches.**

Several categories emerged from the data, with the variation in these categories determining which approach adults followed. Dieting, as described by several adults, involved following either an established diet (e.g., Israeli Army Diet or Air Hostess Diet), a commercial weight loss program (e.g., Weight Watchers or Jenny Craig), or liquid diet supplements (e.g., Tony Ferguson) to lose weight and become slimmer. In contrast, the non-dieting approach was popularly termed ‘lifestyle change’ by several adults, and referred to a self-imposed approach primarily focused on increasing quality of life and longevity by adopting a healthier lifestyle, without following an established diet. There was overlap to some degree as non-dieters sometimes identified weight loss as a secondary goal. Emerging categories which determined dieting and non-dieting attempts included the focus of each approach; attributions about dieting failure; attitudes towards established diets; personal autonomy; perceived knowledge and self-efficacy with an approach; the time perspective of change (e.g., short or long-term), and the perceived identity of the person. Each determinant contributed towards the decision to diet or not diet and this decision was often reinforced by experiences with the approach (see Figure 1). These determinants will be discussed in turn, and will be illustrated with quotes which attempt to capture the richness and meaning of this concept. Participants’ identification code, gender, and age are displayed in brackets after these quotes.

### Focus of approach

The focus of each approach differentiated between dieting and non-dieting approaches for some adults. This category ranged along a continuum from a focus almost exclusively on losing weight and body image (determining a dieting attempt) to a broader focus on a healthier lifestyle and integrating health behaviours into daily life (determining a non-dieting approach). Most adults who decided to diet primarily expected weight loss and success in this area reinforced the decision to continue dieting. In contrast, increased quality of life and longevity through gains in physical health were the primary outcomes expected and/or reported on by non-dieting adults, reinforcing the decision to not diet. While weight loss in this approach was still viewed as desirable, it was secondary to lifestyle concerns and seen as a by-product of a healthy lifestyle.

These contrasting motivations were explained by dieters who had transitioned to non-dieting approaches. Initially when dieting, decisions were sometimes based around how much weight would be lost, in contrast with measuring how much healthier a choice might be. Dieting approaches sometimes overemphasised weight and underemphasised health:

“Weight loss (was my first attempt to change) like I was living to lose weight and the choices I was making were not ‘I want to lead a healthier life, I want to change my life and my lifestyle’. Or at least recognising that to be a healthier weight, to be physiologically healthier and a better weight for my back and all those sorts of things, I wasn’t acknowledging those sorts of things. Previously it was: ‘I want to be thinner’.” [Participant 4 – Female – 35]

In the middle of this continuum, diets were contrasted with lifestyle change, but weight loss was still a focus of the approach:

“I don’t think it is diet I think it is a lifestyle change. I think if you are happy within yourself and you have got a full life, that weight loss will come all so naturally as well, it has for me” [Participant 13 – Female – 41]

Dieting appeared to be strongly associated with losing weight, and needing to lose weight was sometimes based on image for a certain event rather than for health reasons. Decisions to diet were reinforced by previous weight loss success through following this approach:

“It (dieting) certainly taught me how to lose weight if I needed to and I was able to put that to good use before I got married and stuff… nobody wants a fat bride…” [Participant 1 – Female – 51]

A desire to balance quality of life in different life domains (e.g., relationships & physical health), accompanied by resignations that weight loss might be more difficult, contributed to the decision to follow a non-dieting approach. One example of this was the reluctance to compromise on dinner dates with family and friends:

“I definitely like to go out for dinner a couple of times a week. That’s part of my relationship with my friends and my husband so I’m not prepared to just never eat out. So whatever weight I can get to without sacrificing those things, fine.” [Participant 4 – Female – 35]

The decision to adopt a non-dieting approach was also motivated by a desire to focus on slowly integrating health behaviours into one’s lifestyle. More clarity on the subtle nuances between dieting and non-dieting approaches assisted this integration:

“I think focusing on weight is the fool’s errand, because what, really, it can’t be the measure, because what does a lower number on a scale really give you? It doesn’t give you anything in my mind. What gives you something is being healthier and part of the eating better.. like now for me I’ve taken up running heaven help me, and if I’m not eating the right things I can’t run well.” [Participant 4 – Female – 35]

### Attributions about dieting failure

Attributions about dieting failure also emerged as a category which appeared to differentiate between dieting and non-dieting approaches for some adults. Some dieters internalised failure and emphasised the importance of persisting in the face of letdowns, an attitude which contributed to successive dieting attempts. Persistence appeared to be a natural response to failure:

"“At the time (after a failed weight loss attempt) I feel like a failure, but I also know that you don’t fail until you give up. And if I am 99 before I bloody get to my goal weight I am not going to give up.” [Participant 7 – Female – 48]"

Failures were sometimes attributed to laziness and poor self-discipline and were discussed in terms of personal responsibility. A disciplined approach to restriction was seen as preventing laziness and overconsumption:

“I couldn’t maintain the discipline to stay hungry…when I needed to be hungry to lose weight… well 6 sessions over 3 months and no net weight loss so I didn’t go back to see her so yeah there needed to be a lot of discipline… and if you don’t feel like being strict with yourself about the quantities of food you end up being lazy and eating more… I did I should take responsibility for that, I did.” [Participant 3 – Female – 56]

In contrast, when others reflected on dieting attempts, some of the failure was attributed to the diets they had been trying. Some participants became sceptical about the value of diets and resolved to adopt a non-dieting approach in light of their failures. A strong association between dieting, fatness, and negative emotions was formed which appeared to contribute towards a critical appraisal of their dieting history and thus an external attribution. In formulating a new approach, participants contrasted this dieting mentality with the behaviour and lifestyle of a healthy person:

“Well I think if you are the diet queen… if this is what dieting does to me then I don’t think it works… here I was 100kgs and fat and miserable and I was just always on diets, so I thought, well, maybe it’s just not me that’s the problem, maybe it is the diets as well. So instead of just taking complete ownership that I was a failure and that I was weak and couldn’t stick at things I decided to really analyse the way I chose to do it. And that’s when I realised that maybe that was also the thing that was also the big problem, that the dieting was not helping me at all, and that’s when I started having that shift in my subconscious and I think a lot of the time it is the mindset that changes things for you, at the end of the day… I wasn’t going to continue doing it that way I thought, well how do normal, healthy people live?… And started trying to implement it that way instead.” [Participant 12 – Female – 55]

### Attitudes towards established diets

This category ranged from trust to distrust which appeared to determine non-dieting and dieting approaches respectively for some adults. Trust in established diets was demonstrated by a belief in their efficacy and hope in finding a sustainable diet, regardless of the numerous diets on offer and contradictions in the industry:

“And this is also crazy, you know there are so many diets out there, and they all contradict each other. Quite often I go into tears and my head spins… and the thing is everything works, every diet works, but you have to choose what works for you, what is sustainable for you, what you can have in your life. Atkins works and if you read the books they say that it was actually created for his heart patients, and yet you would think eating all bacon and stuff like that would kill you, but cholesterol lowers and weight lowers and everything else lowers, and that works but that is totally out of whack with what Weight Watchers says, and that is totally different to what Tom Dick and Harry say, but they all work.” [Participant 7 – Female – 48]

Other adults acknowledged their scepticism about established diets, based sometimes on conclusions about the commercial motives of the dieting industry and observance of repeated failure in other people attempting these diets. This attitude led to rethinking dieting attempts and adopting what was perceived as a more sustainable, non-dieting approach:

“All the diets out there I think give you only half the information because they make their money from you not achieving. So once you understand that that is the commercial world that you live in, I know people who have done those diets, they have lost weight, they have put it on, lost it, and put it all back on again. I probably know 6 people personally that that is what they have gone through. And I didn’t want to put in all the effort of losing the weight only to turn around and put it all back on once we are married sort of thing.” [Participant 10 – Female – 37]

Lodged between these two perspectives were adults who affirmed the philosophy of non-dieting approaches (e.g., enjoying lifestyle behaviours) but also believed in the efficacy of diets and continued dieting. This was despite an acknowledgement that diets were not the answer to sustained weight loss:

“I know a lot of people go on about how they are on a diet, and once they lose the weight they will be right. But it’s not like that it needs to be an entire lifestyle change (to sustain weight loss), and you need to fall in love with the healthier lifestyle change so it’s not an effort it needs to be just what you want to do. And that is it pretty much.” [Participant 7 – Female – 48]

### Personal autonomy

The personal autonomy surrounding the decision to diet or not diet also appeared to influence which approach would be taken. At one end of the spectrum, some adults who decided to diet described the influence of others in this decision. In the following example, multiple dieting attempts were attributed to socialisation into this approach at a young age. Weight was considered a multi-generational issue in the family, and despite negative experiences and counterintuitive results (e.g., weight gain) initially, early exposure was viewed as the trigger for successive dieting attempts. Weight loss formed a strong association with success, worthiness, and acceptance within the family circle:

“My mother, who had weight issues of her own, dragged me along to Weight Watchers for the first time. But of course I didn’t really want to go so, rather than losing weight, I actually gained it. But that sort of started the diet yo-yo thing. And you name the diet I have done it. My parents are highly highly critical and they only ever complimented me about 3 years ago on one thing I have ever done, and I feel that everything I have ever done, my weight is my only thing that I haven’t succeeded in in every aspect of my life and I feel as if everything I do I am out to prove to them that I am ok, that I am worthy.” [Participant 7 – Female – 48]

In other situations, adults appeared to adopt diets based on more innocuous influences from others. For instance, professional training environments provided one opportunity for adults to be influenced to diet by credible people:

“And the person in charge of that (food preparation area use used by nurses in training) encouraged us to put ourselves on diets if we thought we needed it so I lost quite a bit of weight in there.” [Participant 11 – Female – 66]

Diets were also adopted based on observations and the reports of friends. These diets would be followed if friends had good success with losing weight on the diet:

“I chose this diet because two friends had enormous success; they lost an enormous amount of weight.” [Participant 8 – Female – 61]

Diets were also followed in order to comply with the advice of health professionals. These diets appeared to be focused on losing weight in a short period of time and careful self-monitoring of food intake:

“And going to a dietician and trying really hard for a week or two on this diet and I think I lost a half a kilo or so and getting in trouble because I hadn’t lost more like being told off by this dietician because I hadn’t lost more and you know she was having me count the carrots that I ate and things like that…” [Participant 16 – Female – 38]

Dieters appeared to be more susceptible to follow the recommendations of others than non-dieters in order to gain social approval, based on the perceived acceptability of losing weight and having a certain body shape. This was despite opinions from others disconfirming this perception:

“Even though people have said it doesn’t matter to them, I feel like I would be more likeable, more attractive to people as a general rule. I still think that people think of fat people as being unhealthy, lazy, people, so definitely if I can be slimmer then people would think I would be more healthy as a person, more motivated.” [Participant 13 – Female – 25]

In contrast, non-dieters, while still influenced by others to make changes, sometimes appeared to be more independent in deciding which approach they would choose. On occasions, this occurred after firm words from significant others:

“She (wife) just gave up at one point and said listen, you must do what you want to do, and I will just live with regret of what could have been. They were her very words I remember them very well: ‘so I will just live with the regret of what could have been a very long and happy life together.’ That was one of those things that sort of contributed to me deciding to make changes.” [Participant 19 – Male – 51]

It was also expressed adamantly that a healthy lifestyle was not the default option in the surrounding environment. Instead, a non-dieting approach focused on a healthy lifestyle was emphasised as a clear, conscious, and individual decision that a person needed to make:

“The healthy lifestyle doesn’t happen, our environment, our default position is not a healthy lifestyle. You have to make a clear conscious decision and a very conscious choice to do it and you have got to take action to actually implement it. If you just go with the flow and eat what is put in front of you and what people generally eat in the takeaway culture and the lack of exercise culture and getting in your car to get to work, the default decision is not to be healthy. It’s a conscious choice and an individual choice, not a choice the government or your wife or family can make for you; it is something you have to make for yourself.” [Participant 19 – Male – 51]

### Perceived knowledge and self-efficacy with approach

Knowledge of alternative approaches to dieting also appeared to determine which approach adults would take. Adults who didn’t seem to feel knowledgeable and confident in their ability to make their own eating plans preferred to rely on dieting plans that had previously worked for them:

“Just the diet plans, what they sort of say is a strategic plan. The other strategies work for me, I don’t think I have the knowledge yet to really plan for myself, but after I have been doing it for a while I will be able to plan.” [Participant 14 – Female – 25]

Other adults cited their earlier naivety in believing in shortcuts and confusion from absorbing copious amounts of information. This time was eventually seen to pay off through the knowledge and confidence gleaned from these experiences with dieting. This knowledge and confidence seemed to provide the foundation for adopting a non-dieting approach:

“I wanted to find shortcuts which don’t exist (in following diets). I have suffered from information fatigue and it gets very confusing. I found what was of value after 4 years and I think I have come to a core of knowledge as one learns more but it has certainly served me well. The changes were very simple, I didn’t know how to exercise or eat healthy. I confused rigorous exercise with physical activity which is not the same thing and I confused dieting with healthy eating so the approach I basically took in this was to make small changes, don’t try to be too dramatic about it, just do what the dietician basically tells you and the doctor tells you, and the heart presentation guidelines and stuff, but just do it, and be consistent. So I didn’t go on a diet, I just started making those slow, healthy choices, and started becoming physically more active, slowly but certainly.” [Participant 19 – Male – 51]

### Time perspective

Perceptions of how long behaviour changes would last for also looked to contribute towards the type of approach adults would select. Some adults suggested that diets were chosen with a short-term perspective in mind and viewed as a finite process. Decisions to diet could be reinforced by rapid weight loss, accompanied by the hope that weight would never be put on once reaching a goal weight. Holidays were viewed as a slight interruption to weight loss maintenance:

“Dropping weight and seeing it come off quickly would inspire me to keep going on the diet and get the goal weight. I would hope that after I reach the goal weight I would never put the weight on again. I would expect to put a couple of kilos on when I go on holiday, but then having succeeded, I would hope to jump back into the program and lose those couple of kilos that I had put on.” [Participant 8 – Female – 61]

In the middle of this continuum between short- and long-term perspectives were participants who, while lowering their expectations of weight loss and phrasing their approach as lifestyle change, suggested that this change had happened overnight:

“Instead of expecting great loss every week I am going to be happy with the 500g or up to 2kgs or whatever. So the weight loss is slower for me but not so much the lifestyle change because that happened overnight pretty much after going to the Weight Watchers, and that happened I was like nup (no) I’ve got to make a change so that happened pretty much within 24 hours, so it seems like it’s a lot slower this time.” [Participant 14 – Female – 25]

In contrast, non-dieting approaches appeared to be adopted for an indefinite period of time and were depicted by some adults as a continuous process of changing and improving patterns of eating and physical activity to increase quality of life and longevity. Deciding to follow a non-dieting approach seemed to be reinforced by improvements in physical functioning (e.g., easier to climb the stairs) and quality of life (e.g., less back pain). Some participants emphasised reduction rather than abstinence and began this process by estimating how much restriction they would be prepared to subject themselves to. This was accompanied by an understanding and appreciation that weight gain would result after periods of unsustainable restriction had ended:

“I didn’t immediately go along and say well I’m going to cut everything out, I went ok, and I still believe I’m only going to reduce anything, I don’t believe in low-calorie diets and all the ultra-low-calorie stuff because I’m not prepared to do anything to lose weight that I’m not prepared to do to maintain it, because there is no point in getting there. If I’ve got to go, if I’ve gotta be really stringent in a way that I’m not prepared to live in an ongoing way for the rest of my life then what’s the point in doing that to get to the weight at which I can’t maintain or I am not prepared to live the lifestyle to maintain?” [Participant 4 – Female – 35]

Notably, when some adults were asked about the concept of lifestyle change this elicited negative reactions, due to its association with commercial weight loss programs and observing others who used the term but did not seem to change in the long-term. Everyday actions were perceived to speak louder than words:

“I think conceptually a lifestyle choice is an excellent thing, you make a choice to change your lifestyle. I just reacted badly to it, because of the number of times I have heard it bandied around by people who stuck it. I just think the idea of a lifestyle choice, it is a one and done thing, I make the choice to change my lifestyle, and then forevermore, I will eat healthy and be active. I don’t think it works like that, I think it is a continual choice, it is an everyday choice, it is a busy at work but I am going to go the gym choice. It’s I am feeling peckish, but I am having the better of the two options. I don’t think it is… I guess my issue with the lifestyle choice is that it is a one and done thing. It doesn’t allow for the fact that you have to continually choose that lifestyle every day.” [Participant 21 – Male – 31]

### Perceived identity

Adults’ perceived identities also seemed to indicate whether they would choose a dieting or non-dieting approach. This category varied from a perceived identity as a ‘fat’ person, ‘overweight’ person, or ‘dieter’, to identifying oneself as a ‘healthy person’. A transition from dieting to non-dieting approaches was accompanied by this shift in identity and the use of different labels. This was emphasised by some as a change that happened immediately, with the new identity serving as a reminder of the food-related choices which were appropriate for a ‘healthy person’ to make:

“You know everyone has a title for themselves in life, I am this and I am that, and I was a dieter, whereas I changed in that day and said no I am a healthy person, and it is like a mantra for me, and if you think and act like a healthy person you will become one. So anytime I say to people if you ever thinking well should I eat this or should I do this ask yourself well what would a healthy person do? I mean a healthy person would occasionally eat chocolate and takeaway but they would not have it as part of their routine. I eat healthy, I try and be as active as I can, and I have a mentality that says that I am a healthy person so whatever I do I make the decisions on being that. I am not a fat person I am not a overweight person, I am not a dieter; I am a healthy person and I live that in that scenario.” [Participant 12 – Female – 55]

For some dieters, moving along this identity continuum away from being a ‘fat’ person was perceived as difficult given that they were protected by and familiar with their existing identity. This perception of protectiveness was for some based on protecting their marriage, after observing others who, when losing weight, had apparently encountered difficulty in their marriages. The vulnerability associated with losing weight and being thin also appeared to be a barrier to this transition in identity:

“And I have known a number of women who have lost a lot of weight and their husbands couldn't deal with it and their marriages have broken up. And of course as you lose the weight you become more vulnerable because people see you, you lose the fat identity and they see you for who you really are, and that is scary. It can be scary. Because it is almost as if you are walking around without any clothes on. It's just that protection of while I am a fat person that's what I am known as.” [Participant 7 – Female – 48]

Other adults noted that beliefs about their changing identity were influenced by how other people treated them at different weights. Adjusting from a self-concept described as 'overweight' and 'a bit fat' to 'normal sized' was unfamiliar and perceived as difficult to achieve:

“Yes. Certainly my perception is people will treat you differently at different weights. I think when you are obese you are both more visible and less visible and invisible. And so I think those things translate to self-concept stuff and identity stuff. I think that is my key issue now, identity stuff… and I think that’s why I’m struggling with the last 10 kilos… because it’s going from someone who is still overweight, and still a bit fat, to normal sized, and that’s not a concept I have of myself as being normal sized. It’s a threshold I’m having trouble jumping over.” [Participant 4 – Female – 35]

## Discussion

Participants in this study expressed clear distinctions between the determinants of dieting and non-dieting approaches. Determinants which emerged as themes included the focus of each approach (weight/image or lifestyle/health behaviours); attributions about dieting failure (internal or external); attitudes towards established diets (trusting or distrusting); personal autonomy (low or high); perceived knowledge and self-efficacy with approach (low or high); the time perspective of change (short- or long-term), and the perceived identity of the person (fat/dieter or healthy person).

The focus of approach differentiated between dieters and non-dieters, and ranged from a focus predominantly on losing weight and body image to a broader focus on health and integrating health behaviours into one’s lifestyle to increase quality of life and longevity. This is consistent with the results of other studies. For example, another qualitative study [43] suggested that the identification of health as a motivator for weight loss proceeded from a more functional understanding of health, wherein health was seen as a means to an end to fulfil various social roles and live a long and meaningful life. Health was viewed not in terms of weight but more for its ability to achieve outcomes (e.g., run up stairs or attend work). In contrast, the aesthetic ideal described by author conflated thinness and a healthy weight with health, and may indicate why some adults in our study were overly concerned about these dimensions. A grounded theory study of bloggers on the Fatosphere, an online fat acceptance community [44], also documented a transition from one focus (weight loss and thin ideal) to another (broader focus on indicators of health and well-being) as participants embraced the concept of fat acceptance. Our findings support this existing literature by indicating that this thin ideal precipitates a dieting attempt to lose weight, while the broader focus on health and well-being leads to a non-dieting approached focused more on integrating health behaviours into one’s lifestyle.

Attributions for dieting failure also varied between adults, ranging from internalising dieting failure, which may have motivated some participants’ attempts to persist with diets, to externalising dieting failure, which may have led others to question the effectiveness of diets and adopt a non-dieting approach. Rather than blaming dieting websites for inaccurate or misleading information, some obese adults may blame themselves for not applying dieting information correctly [45]. This internalisation of dieting failure might result from an overemphasis on the health consequences of ‘being fat’ and the accompanying messages of personal responsibility [6], or through moral frameworks provided and perpetuated by diet group leaders and fellow dieters which assign blame and culpability to explain eating behaviours [46]. Feelings of personal failure are also particularly prominent when other friends and family members have had success on commercial diets [35], which the authors noted may reinforce a sense of personal failure. Our findings add to these by that showing that the sense of personal failure may reinforce a dieting attempt so adults are seen as persistent, disciplined, and eventually a successful adherer. We also found that an extended and isolated consideration of this failure with diets can precede adopting a non-dieting approach, which complements the grounded theory [44] suggesting that exposure to fat acceptance materials and challenging the dominant discourse can assist adults in adopting non-dieting tenets.

Attitudes towards established diets appeared to influence whether adults would adopt dieting or non-dieting approaches. These attitudes ranged from trusting and consequently persevering with diets to distrustful and consequently disengaging. Although obese individuals describe feeling exploited by weight loss companies, they feel there is little alternative as other long-term support is perceived to be unavailable [13,35,44]. Ineffective diets are sometimes adhered to because of ongoing support rather than the effectiveness of the diet itself [35]. Our findings add to these by indicating that belief in the efficacy of these diets and the hope of finding a sustainable diet continue to reinforce dieting attempts. In contrast, we found that questioning claims and motives of the dieting industry in the face of a viable alternative led to a non-dieting approach, and have noted identical attitudes in overweight/obese adults blogging on the Fatosphere fat-acceptance community [44]. Notably, this community can also provide support and solidarity for obese individuals who feel stigmatised and socially unaccepted in the wider community [45]. As an example, this support and solidarity might be important for dieters who wish to stop dieting but know of no other options, and non-dieters who wish to find support and acceptance for choosing to focus mainly on health instead of primarily dieting and weight loss.

More autonomous adults appeared to select non-dieting attempts while those influenced by others chose established diets. Obese adults have reported being introduced to diets by their social network, including family, friends, and work colleagues [35]. Adults in this study reported feeling accepted when participating in diet groups, with weight loss success stories of others giving them a sense of hope and encouraging them to diet. Similar to other studies [47], we found that some adults also engaged in weight loss attempts through dieting to prove to family members that they were worthy and acceptable, based on the moral standards held by others about the unacceptability of being fat. Non-dieting adults did not describe other individuals or groups influencing their choice of approach, but recent qualitative research suggests their experiences may be the exception rather than the rule [44]. Bloggers on the Fatosphere fat acceptance community have said it is a “revelation” that there is a community of people (the Fatosphere) that believe: a) fatness is not a personal failing, b) that dieting does more harm than good, and c) that being thin is not a requirement for living a happy and fulfilling life [44]. The authors also noted that the long-term body rejection and intolerance experienced can initially lead to a rejection of non-dieting concepts, which for one participant eventually took more than a decade to consider. Our findings add to these by suggesting that the lack of conformity to social networks (particularly dieting circles) makes some adults well suited to non-dieting approaches, and may be a stepping stone for others on their way to adopting a non-dieting approach. These findings are also consistent with the distinction between *autonomous* and *controlled* motivations encapsulated within the basic psychological need of autonomy originating from Self-Determination Theory [48]. In the context of the theory, autonomy refers to an individual’s desire to organise their own experience and behaviour in accordance with their integrated sense of self [49].

The personal knowledge and self-efficacy with making lifestyle changes gleaned from past dieting experiences contributed towards making an independent decision to adopt a non-dieting approach. More knowledge and self-efficacy contributed to the decision to adopt a non-dieting paradigm, while adults who were not confident about their ability to make their own plan preferred to rely on established dieting plans. Unfortunately, these diets are sometimes not seen as one component of a comprehensive weight loss strategy involving exercising or physical activity [44]. The authors argued that people living with obesity have been ‘socially conditioned’ to turn to diets as a remedy against obesity, and other qualitative accounts indicating that diets are an ineffective but inevitable process [47] would seem to support this. In contrast, fat acceptance is a concept that is “stumbled” across [44]. Collectively these studies suggest that there may be a lack of perceived alternatives to the dieting approach. Our findings add to this literature by indicating that knowledge and self-efficacy with implementing an alternative non-dieting paradigm is one determinant of adopting this approach. These findings correspond to *perceived selfefficacy* (someone’s belief they can attain the goals they set for themselves), originating from Bandura’s Social Cognitive Theory [50].

The time perspective adults held also influenced whether they would choose a dieting (short-term perspective) or non-dieting (long-term) approach. The short-term perspective is evident in previous qualitative studies wherein dieting is viewed by obese adults as an effective way to lose weight in spite of the recognition that diets did not result in sustained weight loss over time [44]. Female weight loss maintainers also noted experiencing a shift in their perceptions about the timeline of health behaviour change, which transitioned from a finite short-term diet mentality towards an ongoing long-term lifestyle change as learning occurred over time [51]. Our findings indicate that these time perspectives can be explicitly adopted at the outset of an attempt to change, which can consequently determine the adoption of a dieting or non-dieting approach. Adults who begin with a long-term perspective and a strong emphasis on sustainability may therefore be good candidates for a non-dieting approach.

An identity as a ‘dieter’ or ‘fat’ person also seemed to be associated with the choice to diet, while seeing oneself as a ‘healthy’ person appeared to precede non-dieting approaches. Constant weight loss pressures can lead to an identity dominated by body hatred, inability to lose weight, and an obsession with weight loss solutions [44]. The authors theorised that transitioning to fat acceptance from this state involved a process of self-negotiation. Outcomes of this approach included a redefinition of self and the meaning of fatness, accompanied by a fear of letting go of the thin ideal. To add support to this recently developed theory, we also found that a process of accepting the self and forming a new dominant identity (that of a healthy person) may have preceded the adoption of a non-dieting approach in some overweight/obese adults. In some adults in our study, there was also a struggle in this transition due to the uncertainty involved with the “new self” and the fear of letting go of the “old self”, an identity that was protective and familiar. One participant suggested that this “fat” identity, which was unattractive to others, protected their marriage, citing several women who lost a significant amount of weight and separated from their husbands who could not adjust to this change. Other previous studies have also suggested that some overweight/obese adults redefine themselves as normal-weight people [52] and healthier people, after experiencing sustained behaviour change in the longer term [51]. Our findings suggest that for some adults, this redefinition may also take place prior to lifestyle change or significant weight being lost and may precede a non-dieting approach.

Several limitations are present in this study. Firstly, participant self-selection biases our study due to an underrepresentation of less educated, male, and overweight perspectives. Secondly, the qualitative methodology and aforementioned sampling procedure preclude making any generalizable statements about the determinants of dieting and non-dieting approaches. Future research could use quantitative methods to investigate these concepts and their relationship with dieting/non-dieting approaches in a larger sample to confirm these relationships. Despite this, our research has several strengths. Firstly, the grounded theory approach ensured the concepts arising from our data were grounded in the perspectives of overweight/obese adults. Secondly, the contrasting determinants of dieting and non-dieting approaches have been relatively unexamined and these findings will assist in understanding who may be best suited to each type of approach. Lastly, the methodology used precluded any apriori assumptions being made about participant experiences, which allows this study to contribute to the growing body of literature giving overweight/obese adults a voice in the treatment of overweight/obesity. Interested readers are referred to two recent qualitative syntheses on weight management [36,53] which capture much of this literature.

The theory emerging from our data suggests that the decision to adopt a dieting or non-dieting approach might depend on several intra- and interpersonal characteristics, including motivational, attitudinal, attributional, autonomous, self-efficacy, time-perspective, and identity-related characteristics. These characteristics all have implications for the framing and subsequent interpretation of public health messages. We suggest that public health messages may point overweight and obese adults in the direction of weight-focused, dieting approaches if they: a) explicitly mention weight and emphasise healthy lifestyle behaviours as a means to weight loss, b) overemphasise moral dimensions, personal responsibility and discipline, c) promote the efficacy of established diets, d) encourage reliance on established programs and significant others without encouraging independent and critical thinking about tailoring treatment, e) promote short-term efforts and goals, and f) distinguish subtly between normal weight and overweight/obese adults in ways that favour normal weight population subgroups.

In contrast, the promotion of non-dieting approaches might stem from public health messages which: a) focus on the multidimensional aspects of health and lifestyle factors rather than weight alone, b) avoid moral discourses and an overemphasis on personal responsibility and discipline, c) empower overweight/obese adults by encouraging information seeking and critical evaluation of different treatment approaches, d) place an emphasis on small, long-term changes, and e) promote healthiness as a trait available to everyone. Public health policy and practice might then focus on delivering consistent messages which reinforce the adoption of one approach over another.

## Conclusions

The model of determinants elicited from this study assists in understanding, from the perspectives and experiences of overweight/obese adults, why dieting and non-dieting approaches are adopted. Both approaches are adopted for contrasting reasons which sometimes overlap (i.e., non-dieters still mention weight as a focus of the approach). Understanding the determinants of each approach will assist clinicians and public health researchers to: a) appreciate why approaches are chosen, b) consider who may be best suited to each approach, and c) encourage adults to adopt one approach instead of the other.

## Competing interests

The authors declare that they have no competing interests.

## Authors’ contributions

SL carried out the design, data collection, analysis and interpretation of data, and drafted the manuscript. ES participated in the design of the study, analysis and interpretation of the data, and critically reviewing the manuscript. X-YH contributed to the design of the study, analysis of the data and to critical revision of the manuscript. All authors read and approved the final manuscript.

## Pre-publication history

The pre-publication history for this paper can be accessed here:

http://www.biomedcentral.com/1471-2458/12/1086/prepub
